# Perturbations in the uterine luminal fluid composition are detrimental to pregnancy establishment in cattle

**DOI:** 10.1186/s40104-018-0285-6

**Published:** 2018-09-17

**Authors:** Thiago Martins, Guilherme Pugliesi, Mariana Sponchiado, Angela M. Gonella-Diaza, Oscar A. Ojeda-Rojas, Frederich D. Rodriguez, Roney S. Ramos, Andrea C. Basso, Mario Binelli

**Affiliations:** 1 Department of Animal Reproduction, School of Veterinary Medicine and Animal Science, University of São Paulo, 225, Avenida Duque de Caxias Norte, Jardim. Elite, Pirassununga, SP 13635-900 Brazil; 2 Department of Nutrition and Animal Production, School of Veterinary Medicine and Animal Science, University of São Paulo, 225, Avenida Duque de Caxias Norte, Jardim. Elite, Pirassununga, SP 13635-900 Brazil; 3 University Corporation of Huila, Prado Aldo: Calle 8, n° 32-49, Neiva, Huila Colombia; 4 ABS Global, 1525 River Road, Deforest, WI 53532 USA; 5 In Vitro Brasil, 340 Rodovia, Km 166 – Soares, Mogi Mirim, SP 13800-970 Brazil; 6 Department of Animal Sciences, University of Florida, Gainesville, FL P.O. 110910 USA

**Keywords:** Albumin, Embryo, Histotroph, Protein

## Abstract

**Background:**

A major, unresolved issue is how the uterine microenvironment determines pregnancy success in cattle. Before implantation, conceptus development depends on the uterine secretome (i.e., histotroph). Despite its pivotal role, little is known about the dynamics of histotroph synthesis and changes in composition throughout the early diestrus and the relevance to pregnancy establishment. We hypothesize that disturbances on histotroph composition affect the establishment of pregnancy. Aim was to disturb histotroph composition at early diestrus and verify the effects on: (Exp. 1) timing to restore its composition; and (Exp. 2) pregnancy rate after multiple-embryo transfer. Estrous cycle of multiparous Nelore cows were synchronized and estrus was considered d 0 (D0) of the experiments. Disturbance was through flushing each uterine horn with 30 mL of DMPBS and collecting the resulting uterine luminal flushing (ULF) on D1; D4; D7; D1 + D4 + D7. Control group remained not-collected. In Exp. 1, ULF was collected on D7.5 from all animals and used for quantification of total protein concentration and abundance of albumin. In Exp. 2, three in vitro-produced embryos were transferred to the uterine horn ipsilateral to the ovary containing the CL on D7.5 and pregnancy was checked on D25 by ultrasound.

**Results:**

In Exp. 1, ULF collection on D4 or D7 increased (1.5- to 2.2-folds) the total protein concentration and albumin abundance. ULF collection on D1 did not alter (*P* > 0.10) these endpoints. In Exp. 2, ULF collected on D4 or D7 decreased pregnancy rates to approximately half of that measured in the remaining groups.

**Conclusions:**

Subtle perturbations imposed to the native intrauterine milieu, such as those caused by a single, low-volume collection of ULF, profoundly disturbs intrauterine composition and pregnancy success. At least 4 d were necessary for the uterus to recover its composition and the functional capacity to carry post-implantation gestation.

## Background

In eutherian mammals, there are two manners for providing nutrients to the conceptus, histotrophic and hemotrophic nourishment. In ruminants, the histotrophic mode is of special importance, because preimplantation embryo development takes approximately 20 d [1, 2]. During the preimplantation period, the bovine embryo development occurs independently of a direct connection to the blood supply. Development relies solely on a complex fluid secreted by the epithelium lining the reproductive tract (i.e., oviductal and uterine fluid) to supply basic energy needs [1, 2]. Specifically, the fluid secreted into the uterine lumen, termed histotroph, is a complex mixture of growth factors, hormones, enzymes, transport proteins, ions, lipids, glucose, amino acids and other molecules, that are dynamically synthesized by the endometrial glandular and luminal epithelia as well as selectively transported from blood [2–4]. In cattle, the establishment of pregnancy is dependent on the presence of an elongated conceptus, capable of signaling to the endometrium, mainly through interferon-tau (INFT) that is released in increasing amounts from d 15-16 of gestation [2, 5]. The IFNT acts on the endometrium and inhibits the biosynthetic cascade of prostaglandin F_2_α and thereby luteolysis. Conceptus signaling together with continuing P4 exposure further induces endometrial differentiation to support receptivity to the conceptus and implantation. Thus, the histotroph is pivotal for the maintenance and development of preimplantation conceptus in cattle, allowing the establishment of pregnancy.

There is convincing evidence that the phase of histotrophic nutrition is critical for the establishment of pregnancy in cattle. About 2/3 of the overall embryonic death loss (~ 30%) occurs between d 8 and d 16 post-insemination [6–8]. Part of the elevated embryonic mortality that occurs during the preimplantation period is deemed to result from a functional incapacity of the uterine luminal milieu to properly support conceptus survival and elongation.

In fact, the importance of histotroph for the periimplantation conceptus development and survival was nicely demonstrated by Gray et al. [3, 4] using an ovine uterine gland knockout model. The authors verified that the absence of endometrial gland secretions in sheep uterus compromises conceptus survival and elongation, resulting in impaired conception.

The histotroph composition is temporally-regulated by estradiol (E2) and progesterone (P4), along the estrous cycle. In effect, the endometrium undergoes marked functional changes in response to the ovarian-endocrine stimulus and to pregnancy, as demonstrated in transcriptomic studies [9–11]. Furthermore, studies in cattle [12–15] indicated that the histotroph composition also undergoes dynamic changes along the estrous cycle and is altered by different P4 concentrations and pregnancy. Accordingly, proteomic [12, 13], amino acids [14–16] and components of the redox system [17] were contrasting between uterine fluids recovered across the diestrus. The expression of a number of proteins during the cycle was related to P4 concentrations from d 3 to d 7 post-estrus [12] or it was increased when P4 concentrations were exogenously elevated [14, 15]. Altogether, these findings support the notion that histotroph composition results from dynamic changes in endometrial function, orchestrated by sex-steroids and the embryo/conceptus, to provide optimal uterine environments that support stage-specific requirement of embryo/conceptus development. Evidence for this well-regulated environment might be verified by a stringent requirement of synchrony between the developing embryo and the recipient (approximately 24 h) for optimal pregnancy rates [18, 19] in cattle. Indeed, transfer of a single d 7 embryo towards a synchronous d 7 uterus resulted in greater pregnancy rate compared to transfer towards an asynchronous uterus [20]. Specifically, transfer of a single d 7 embryo to a d 5 or d 8 uterus moderately impacted pregnancy rate (~ 6% reduction), but transfer of a single d 7 embryo to a d 4 or d 9 uterus had negative impact on pregnancy rate (~ 22% reduction). In addition, indirect modifications introduced in the uterine environment, for example as caused by supplementation of P4 at early diestrus, advances conceptus elongation [21] and influences embryonic survival and the subsequent fertility [22]. However, the dynamics of histotroph changes along the estrous cycle are largely not known. Furthermore, understanding how and to which extent disturbances in this native environment influence pregnancy establishment in cattle is limited. Thus, in the present study we aimed to disturb the histotroph composition at early diestrus and verify the effect on 1) timing to recover composition and 2) embryo survival in beef cattle.

## Methods

### Animals

Non-lactating, cycling, multiparous Nelore cows (*Bos taurus indicus*, average body weight 593.80 ± 12.07 kg) were maintained under grazing conditions, supplemented with chopped sugarcane, concentrate and minerals to fulfill their maintenance requirements and received water ad libitum. The experiment was conducted in the Southern Hemisphere tropics at the Pirassununga Campus of the University of São Paulo, Brazil. All experimental procedures involving animals complied with the Ethics and Animal Handling Committee of the School of Veterinary Medicine and Animal Science of the University of São Paulo (CEUA-FMVZ/USP, No. 9585220316).

**Fig. 1:**
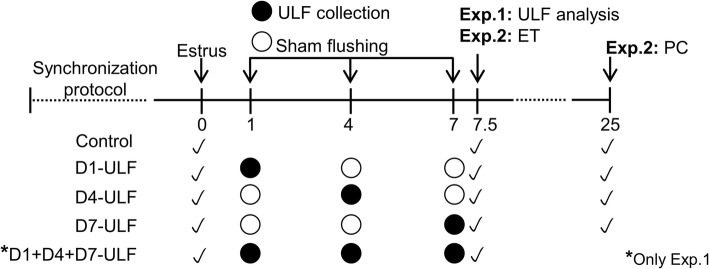
Experimental designs of the two experiments performed in this study. Collection of uterine luminal flushings (ULF) was performed during diestrus to determine: the effect on the uterine luminal protein content at D7.5 post-estrus (Exp. 1) or on pregnancy establishment (Exp. 2). Accordingly, follicle growth of cycling, non-suckled, multiparous Nelore cows was synchronized and estrus was detected (D0). Uterine horns were flushed non-surgically with 30 mL of DMPBS thrice (Exp. 1, 90 mL total) or once (Exp. 2) and ULF was collected. In Exp. 1, animals were submitted randomly to collection of ULF on D1; D4; D7; D1, 4 and 7; or to remain not-collected, to compose the 5 experimental groups: D1-ULF, D4-ULF, D7-ULF, D1 + D4 + D7-ULF and Control. On D7.5, ULF were collected from all groups for determination of total protein concentration and abundance of albumin. On Exp. 2, the same experimental groups were present, with the exception of D1 + D4 + D7-ULF group*. On D7.5, three in vitro-produced embryos were transferred non-surgically to the uterine horn ipsilateral to the ovary containing a CL and pregnancy was checked (PC) on D25 by transrectal ultrasonography. In both experiments, a sham ULF collection (all procedures except delivering DMPBS to the uterus) was performed in each cow, in each experimental day when no ULF collection was scheduled

This study comprises two experiments. In the Exp. 1, we aimed to determine the effect of uterine flushings performed over diestrus on the uterine luminal protein content at d 7.5 post-estrus. In the Exp. 2, the aim was to determine the impact of uterine flushing on the establishment of pregnancy in embryo recipient cows. The experimental designs are illustrated in Fig. 1.

### Experimental designs

In the Exp. 1, follicular growth was synchronized using an intravaginal P4-releasing device (1 g; Sincrogest®, Ourofino Saude Animal, Cravinhos, SP, Brazil), an intramuscular administration of estradiol benzoate (2 mg; Sincrodiol®, Ourofino Saude Animal) and prostaglandin F_2_α analogue (PGF_2_α; 500 μg of sodium cloprostenol; Sincrocio®, Ourofino Saude Animal). Eight days later, the P4-devices were removed; cows received another injection of PGF_2_α analogue and an Estrotect™ (Western Point Inc., Apple Valley, MN) heat detector patch. Cows were checked for signs of estrus twice a day between 36 h and 96 h after P4-releasing device withdrawal. Cows observed in standing estrus and/or presenting an activated heat detector patch were considered in estrus (D0 of the study; *n* = 44). Animals were submitted randomly to collection of uterine luminal flushings (ULF) on D1; D4; D7; D1, 4 and 7; or to remain not-collected, to compose the 5 experimental groups: D1-ULF (*n* = 9), D4-ULF (*n* = 9), D7-ULF (*n* = 9), D1 + D4 + D7-ULF (*n* = 9) and Control (*n* = 8). On D7.5, ULF were collected from all groups for determination of total protein concentration and abundance of albumin. Control group was used to assess the perturbations promoted by the ULF collections on these endpoints.

In the Exp. 2, estrous cycles were synchronized using a slightly modified version of the protocol described above. An injection of GnRH analogue (10 μg of buserelin acetate; Sincrofort®, Ourofino Saude Animal) was administered intramuscularly at P4-device insertion, followed by its removal 7 d later and by an administration of PGF_2_α analogue 24 h earlier. Cows were checked for signs of estrus as described previously. Animals observed in standing estrus and/or with an activated Estrotect patch (*n* = 64) were assigned randomly to one of four groups: (i) Control (*n* = 16), (ii) D1-ULF (*n* = 15), (iii) D4-ULF (*n* = 17), (iv) D7-ULF (*n* = 16).

### Ultrasound examinations

Transrectal ultrasonography (Mindray M5, Shenzen, China; equipped with multifrequency linear transducer set to 7.5 MHz) in B-mode was performed to check ovulation of pre-ovulatory follicle on D1 and confirm the side of corpus luteum (CL) on D4 and D7. In Exp.1, diameter of pre-ovulatory follicle and CL area were recorded for analysis. In addition, blood flow of measured CLs was examined using ultrasound Color Doppler-mode, to confirm CL functionality, as defined by Pugliesi et al. [23].

### Quantification of P4 concentrations

In Exp. 1, blood samples were collected on D7 for determination of serum P4 concentrations. Serum was harvested on D7 by centrifugation of blood at 2,700 × *g* for 15 min at 4 °C. Progesterone was assayed using a solid-phase RIA kit (Immuchem™ Double Antibody Progesterone Kit; Cat. 07-170105, MP Biomedicals, NY, USA). The sensitivity of the assay was 0.1 ng/mL. The intra-assay coefficient of variation (CV) for quality control samples was 1.15% (low standard) and 0.01% (high standard).

### ULF collection

**Fig. 2:**
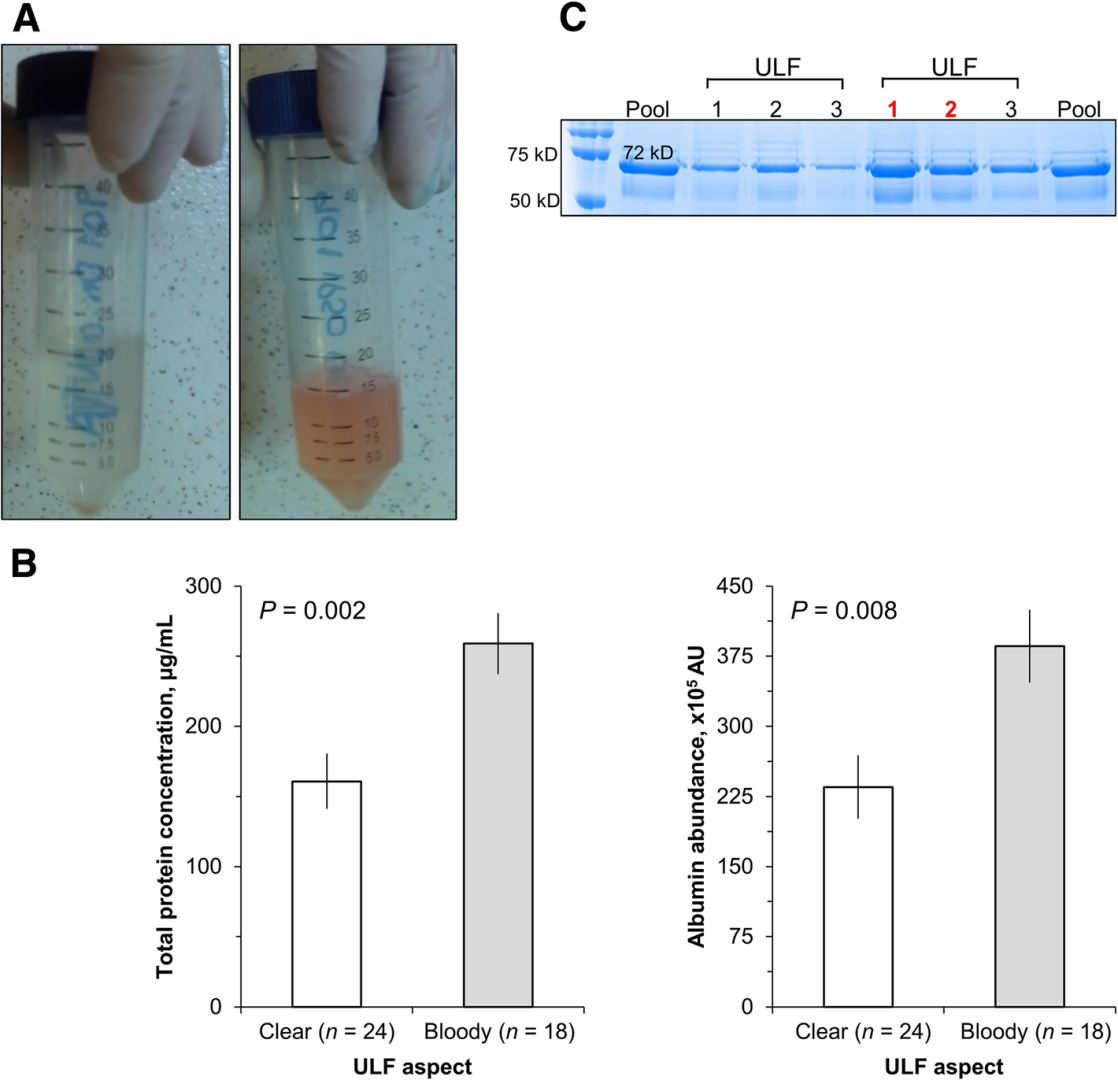
Effect of ULF classified as clear or bloody on total protein concentration and albumin abundance. On D7.5 (D0: estrus), each uterine horn of beef cows (*n* = 7) were flushed thrice with 30 mL of PBS and the resulting uterine luminal flushings were collected (ULF1, ULF2 and ULF3), visually classified as clear or bloody (Panel **a**) and stored for analysis. Quantification of total protein concentrations and determination of albumin abundance was performed in each ULF (Panel **b**). Panel **c**: Representation of the three successive ULF classified as clear (black bold marked) or bloody (red bold marked) in the polyacrylamide gel stained with Coomassie-Blue for albumin determination. Pool: Mixture of all collected ULF. AU: Arbitrary units. Values are presented as LSMEANS±SEM

Caudal epidural anesthesia was performed with 2% lidocaine solution (4 mL; 80 mg; Lidovet®, Bravet, Engenho Novo, RJ, Brazil) immediately before ULF collection. Uterine horns were flushed non-surgically, always flushing the horn ipsilateral to ovary containing the CL first and flushing the contralateral horn subsequently, at different d post-estrus, as described in the experimental design. ULF was collected using a similar method described previously [12]. Accordingly, a sterile silicone Foley catheter (2 vials, 20 mL cuff, 18″ or 20″ diameter; Rusch®, Teleflex, US) was fixed onto a stylette. Guided by rectal palpation, the catheter was passed through the cervix into the body of the uterus and directed to the target uterine horn. The catheter was positioned and fixed at the horn’s bifurcation by inflating the cuff. The distal end of the uterine horn adjacent to the uterotubal junction was manually occluded through rectal palpation. Then, the uterine horn was slowly filled with 30 mL of Dulbecco’s modified phosphate buffered saline (DMPBS, Nutricell, Campinas, SP, Brazil) at 37 °C using a sterile 60-mL catheter tip syringe (SR, Manaus, AM, Brazil). After infusion of DMPBS, the ULF was immediately recovered by aspiration with a syringe only while a steady flow could be achieved. In Exp.1, for each uterine horn, this procedure was repeated three times without removing the catheter. ULF recovered from each uterine horn was placed in 50-mL conical tubes to record volume and visual aspect. ULFs were classified subjectively as bloody when they contained any traces of blood or presented a pinkish coloration, as opposed to a translucent aspect (Fig. 2a). Total volume recovered and visual aspect of ULFs were used to characterize the pooled three ULF collected from each cow. In the groups D1-ULF, D4-ULF and D7-ULF, a sham flushing (all procedures except delivering DMPBS to the uterus) was performed on each experimental day when no collection was scheduled. For example, group D1-ULF was subjected to sham flushing on D4 and D7. On D7.5, each uterine horn of each animal was submitted to a single ULF collection with 30 mL of DMPBS and the ULF was used for protein analysis. Volume and visual aspect of ULF-D7.5 were recorded. In addition, to evaluate the impact of three sequential collections on ULF composition, on D7.5, only in the Control group, two additional collections were performed. Volume and aspect of each ULF collected (ULF1, ULF2 and ULF3) were recorded individually for analysis. On D7.5, ULFs were transferred to light-safe 50-mL conical tubes and kept on ice until processed in the laboratory. Within 15 min of flushing, ULFs were clarified by centrifugation (1,000 × *g* for 10 min at 4 °C), supernatants were transferred to cryotubes and stored at − 20 °C for subsequent analyzes.

Collection of ULF in the Exp. 2 was performed as described, but with minor modifications. Specifically, the uterine horns were flushed only once with 30 mL of DMPBS, instead three times as in Exp. 1.

### Embryo production and embryo transfer procedures

On D7.5, all animals from Exp. 2 were submitted to embryo transfer. Cumulus oocyte complexes used to produce embryos were aspirated from ovaries collected in a local slaughterhouse. Embryos were produced according to a standard protocol for in vitro embryo production [24]. Recipients received a caudal epidural anesthesia immediately before the procedure of embryo transfer. Three in vitro-produced, grade 1 blastocysts were transcervically placed in the middle of the cranial third of the uterine horn ipsilateral to the ovary containing a CL, using standard nonsurgical techniques. By transferring three embryos per recipient cow, we expected to minimize the random effect of a single incompetent embryo to influence pregnancy establishment [25].

On D25, pregnancy status was based on visualization of the embryo proper and heartbeat by transrectal B-mode ultrasonography under optimal conditions, as described previously [26, 27]. All cows received a luteolytic dose of PGF_2_α after pregnancy diagnosis.

### Total protein quantification assay

The protein content in the ULFs collected on D7.5 was determined by a colorimetric assay using a Micro BCA Protein Assay Kit (Pierce Biotechnology, Rockford, IL) according to manufacturers’ instructions. The micro-assays were conducted in 96-well plates. The total protein concentration in each sample was determined in triplicates and calculated using the standard curve supplied on the kit. The standard curve was based in bovine serum albumin diluted in DMPBS and ranged from 25 to 2,000 μg/mL. The colorimetric absorbances were measured by a spectrophotometer (Multiskan MS Primary EIA, Thermo-Fisher) adjusted at 570 nm wavelength.

### Determination of albumin abundance

Albumin content was determined in ULF samples by Comassie-Blue staining. ULF samples (18 μL) from D7.5 were diluted in 2× Laemmili buffer and denatured at 95 °C for 5 min. Proteins were separated in 12% SDS-Polyacrylamide gel electrophoresis at 160 V for 120 min. The resulting polyacrylamide gels were fixed by submerging in 50% MeOH, 10% HoAC and 40% distilled water (*v*/*v*) solution for 30 min. Fixed gels were stained in 0.25% (*w*/*v*) Coomassie Brilliant Blue R-250 (Bio-Rad laboratories) solution for 2 h and then, cleared overnight in a destaining solution (5% MeOH, 7.5% HoAC, 87.5% distilled water). Stained polyacrylamide gels were exposed to ChemiDoc MP Imaging System (Bio-Rad) apparatus and band intensities were quantified by densitometry using Image-Lab software version 4.01 (Bio-Rad). Protein molecular weights were estimated according to a molecular weight standard (Precision Plus Protein™ Dual Color Standards, Bio-Rad laboratories) that was run in each gel. The identification of the albumin band was based on molecular weight of bovine serum albumin (~ 67 kDa). Only ULF samples collected from the ipsilateral uterine horn were used for determining the abundance of albumin on D7.5. The effect of three sequential flushings on the abundance of albumin was determined on ULF collected from the Control animals on D7.5 from ipsi- and contralateral uterine horns.

### Statistical analysis

Dataset of Exp.1 (i.e. total protein concentration and abundance of albumin) was analyzed by ANOVA using MIXED procedure of SAS (SAS Inst. Inc., Cary, NC, USA) version 9.3. Model included fixed effect of experimental group, horn (ipsi- and contralateral) and their interaction. Assumptions of normality of residues and homogeneity of variances were checked by influencing diagnostics outputs from the MIXED and *F*-max test, respectively. Data that did not follow the assumptions were transformed by the natural logarithmic or square root before analyses. When necessary, comparisons were performed contrasting D-ULF groups versus the Control group, using the DIFF command incorporating the Dunnett test.

Aiming to determine the effect of sequential ULF collections on the protein and albumin quantity of collected ULFs, three-sequential ULFs collected on D7.5 were analyzed by split-plot design using MIXED procedures. Model included fixed effect of ULF collection order (ULF1, ULF2 or ULF3), ULF visual aspect, horn and all their interactions. The random effect of cows nested within combination of flushing aspect and uterine horn side was used as an error term to test the main plot effect. The type of variance–covariance structure used was chosen based on smaller magnitude of the corrected Akaike’s information criterion (AICC). When the effect of a categorical variable was significant, the LSD *post-hoc* test was used to compare means.

The variable, volume on D1, D4 and D7 from groups D1-ULF, D4-ULF and D7-ULF, respectively, was analyzed using MIXED procedure. Similarly, binomial variable, proportion of ULF with blood from each group was analyzed using GLIMMIX procedure. Model included fixed effect of group, horn and their interaction. For the D1 + D4 + D7-ULF group, variables volume and proportion of ULF with blood on D1, D4 and D7 were analyzed as split-plot design, including fixed effect of day, horn and their interaction. The random effect of cows nested within uterine horn side was used as error term. On D7.5, those variables (volume and visual aspect) were analyzed including fixed effect of group, horn and their interaction. In addition, volume of three-sequential ULFs collected on D7.5 from Control group was analyzed as split-plot design. Model included fixed effect of flushing order, horn and their interaction.

In Exp. 2, the analyzes of frequency of pregnant cows were performed with FREQ procedure using Chi-square distribution. Orthogonal contrast comparison was used to assess differences between groups (Control vs. ULF-D1, ULF-D4 vs. ULF-D7 and Control & ULF-D1 vs. ULF-D4 & ULF-D7).

Continuous variables are reported as Least Square Means ± Standard error of the mean (LSMEANS ± SEM) and binomial variables as means.

## Results

### Exp. 1

#### Ovarian characteristics

Mean ± SEM of size of pre-ovulatory follicle (13.42 ± 0.27 mm), and CL area (2.42 ± 0.37 cm^2^) and serum P4 concentrations on D7 (3.29 ± 0.24) were similar among groups (*P* > 0.10; data not shown). In all cows, a functional CL was detected on D4 and D7 at ultrasound scan, according to criteria described previously [23].

#### Effect of same day, sequential ULF collections on ULF composition

For this analysis, only data from the sequential collections of ULF on D7.5 from the Control group were used (Fig. 2). We expected that ULF collection would have the effect of reducing total protein concentration and abundance of albumin progressively from ULF1 to ULF3. However, these endpoints were not affected by sequential collections among ULF1, ULF2 and ULF3 (*P* > 0.1). Irrespective of order of collection and side of uterine horn of the collected ULF, compared to the ULF classified as clear, those classified as bloody presented greater total protein concentration (bloody: 258.95 ± 21.97 vs. clear: 160.80 ± 19.55 μg/mL) and abundance of albumin (bloody: 385.78 ± 38.96 vs. clear: 235.06 ± 33.74 arbitrary units; *P* < 0.01).

#### Effect of different days, sequential ULF collections on same day ULF composition

For this analysis, we used ULF samples collected on D1, D4 and D7, from group D1 + D4 + D7-ULF only. There was an effect of day (*P* ≤ 0.05) on the volume of recovered ULF, regardless of uterine horn side flushed. Volume of recovered ULF on D4 (81.8 ± 1.71 mL) was lower than that on D7 (87.7 ± 1.77 mL), but similar to D1 (85.3 ± 1.77 mL). Proportion of ULF classified as bloody for the D1 + D4 + D7-ULF group was not affected (*P* > 0.1) by day nor uterine horn side (D1: 29% [5/17]; D4: 29% [5/17] and D7: 11.1% [2/18]).

#### Effect of different days, single ULF collections on same day ULF composition

Initially, we analyzed volume recovered and visual aspect of ULFs collected on D1, D4 or D7. Because blood flow to the uterus decreases from metestrus to early diestrus, we expected that frequency of ULF containing blood would also decrease from D1 to D7. There was no effect of group (D1-ULF, D4-ULF and D7-ULF) on the proportion of ULF classified as bloody (46.3% [25/54], *P* > 0.1), neither on total volume of recovered ULF (87.7 ± 0.76 mL, *P* > 0.1), regardless of uterine horn side collected.

#### Effect of single or multiple days ULF collections on subsequent ULF composition

**Fig. 3:**
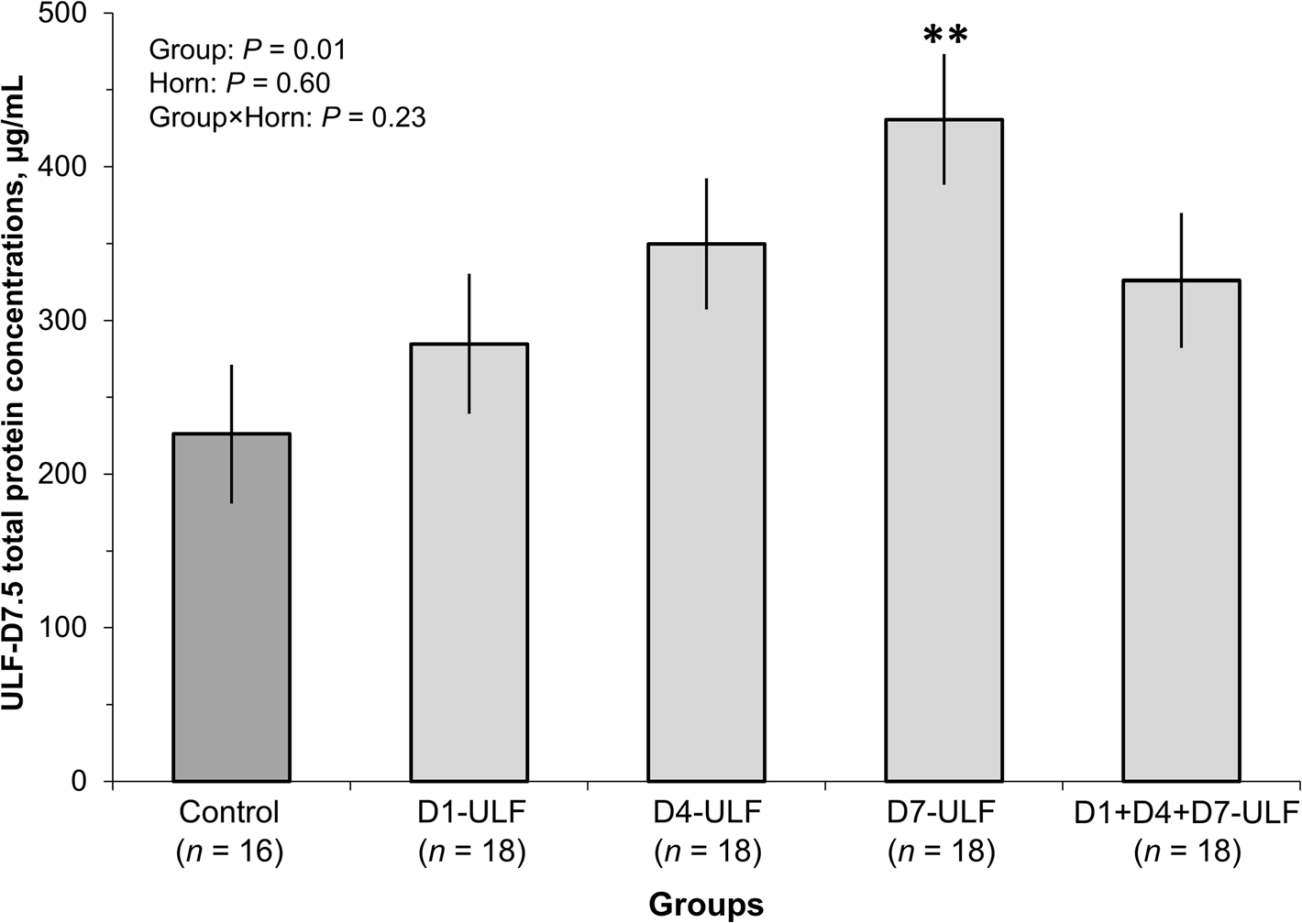
Effect of previous ULF collections on total protein concentration in the ULF-D7.5. Uterine horns of beef cows detected in estrus (D0) were flushed non-surgically thrice with 30 mL of DMPBS (90 mL) and the resulting uterine luminal flushing (ULF) was collected. Animals were assigned randomly to be submitted to ULF collections on D1; D4; D7; D1 + D4 + D7; or to remain not-collected, composing 5 experimental groups. On D7.5, ULF were collected from all groups for quantification of total protein concentrations. Control group was used to assess the perturbations promoted by the ULF collections on the total protein concentrations. Values are presented as LSMEANS ± SEM. Main effects of group, uterine horn (Horn) and their interaction are indicated. **P < 0.01, differed from Control group as determined by Dunnett test

**Fig. 4:**
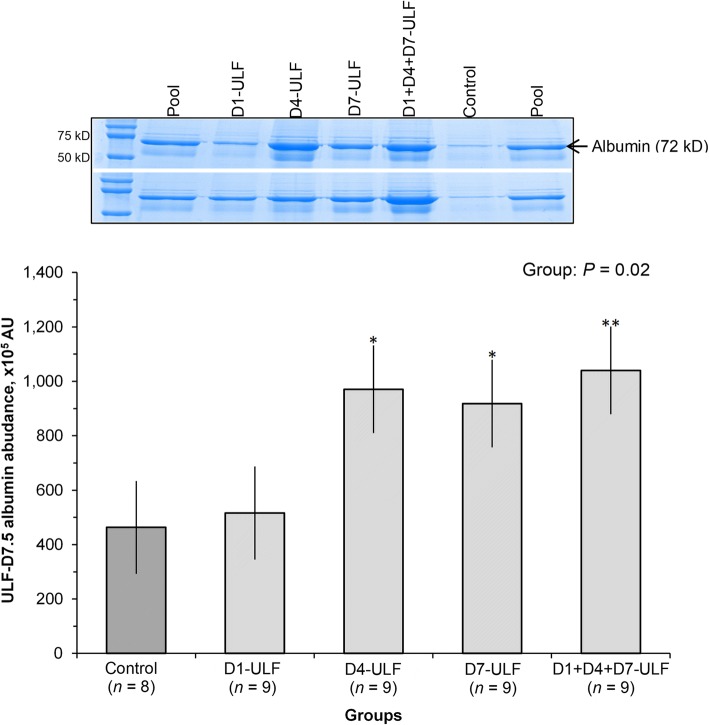
Effect of previous ULF collections on total protein concentration in the ULF-D7.5. Uterine horns of beef cows detected in estrus (D0) were flushed non-surgically thrice with 30 mL of DMPBS (90 mL) and the resulting uterine luminal flushing (ULF) was collected. Animals were assigned randomly to be submitted to ULF collections on D1; D4; D7; D1 + D4 + D7; or to remain not-collected, composing 5 experimental groups. On D7.5, ULF were collected from all groups the abundance of albumen was determined in samples from ipsilateral uterine horn. SDS-PAGE was conducted, polyacrylamide gels were stained with Coomassie-Blue and abundance of albumin was determined by densitometry. Values are presented as LSMEANS±SEM. Main effect of group is indicated (*P* = 0.02). Mean differed from Control at **P = 0.03 and *P = 0.06 as determined by Dunnett test. Pool: Mix of all flushing sample. AU: Arbitrary units

Next, we analyzed ULF samples collected on D7.5 from animals submitted to UFL collections conducted on D1, D4, D7 or D1, D4 and D7. Proportion of ULFs classified as bloody (47.8% [43/90]) and volume collected (26.1 ± 0.53 mL) were all similar among groups (*P* > 0.1), regardless of uterine horn side sampled. Despite these similar gross characteristics among groups, total protein concentration in the ULF-D7.5 was affected (*P* = 0.01) by group, regardless of the uterine horn side sampled (Fig. 3). The D7-ULF had 1.9-fold higher (*P* < 0.01) total protein concentration in the ULF-D7.5 compared to the Control group. Compared to the Control group, total protein concentrations in ULF-D7.5 of groups D1-ULF, D4-ULF and D1 + D4 + D7-ULF increased numerically 1.3, 1.5 and 1.4 fold, respectively, but concentrations were not significantly different than the Control group. Consistently, the abundance of albumin (that was only analyzed in the ULF-D7.5 collected from the ipsilateral uterine horn), there was an effect of group (*P* = 0.02) on the abundance of albumin measured in the ULF-D7.5 (Fig. 4). The albumin abundance in the D4-ULF and D7-ULF groups were respectively, 2.0- and 2.1-fold greater compared to the Control group (*P* = 0.06), while that of the D1 + D4 + D7-ULF group was 2.2-fold greater (*P* = 0.03) than the abundance verified in the Control group. Changes in total protein and albumin abundances were caused by the ULF collection, not by the manipulations associated with the ULF collection. Specifically, compared to Control, the ULF-D1 group had no significant alterations in total protein and albumin abundances, despite of sham flushing procedures performed on D4 and D7.

### Exp. 2

#### The effect of single ULF collections on pregnancy rate of embryo recipients

**Fig. 5:**
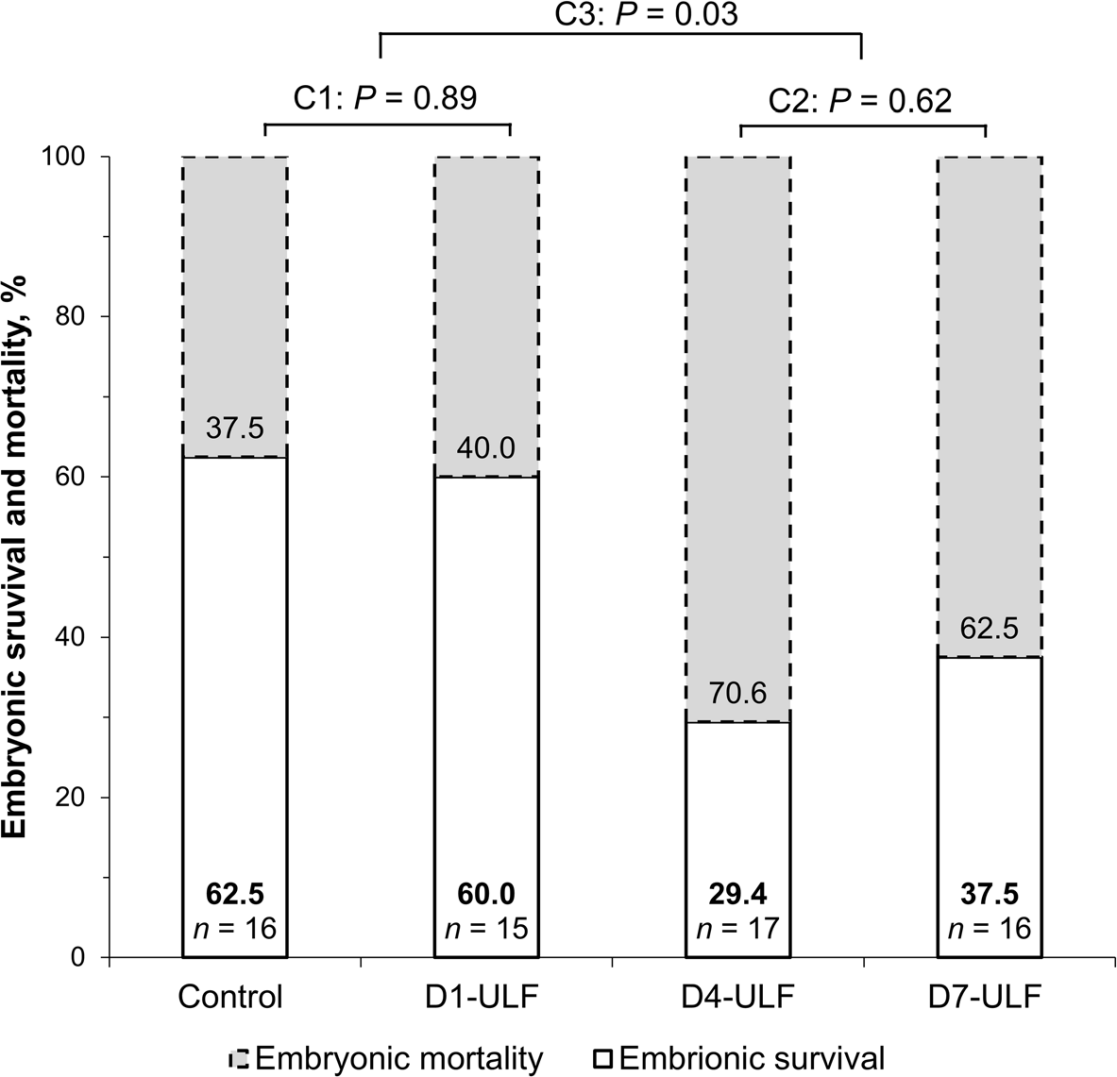
Effect of previous ULF collections on embryonic survival and mortality. Uterine horns of beef cows detected in estrus (D0) were flushed non-surgically with 30 mL of DMPBS and the resulting uterine luminal flushing (ULF) was collected. Animals were randomly assigned to be submitted to ULF collections on D1; D4; D7; or to remain not-collected, composing 4 experimental groups. On D7.5, three in vitro-produced, high-grade embryos were non-surgically transferred to the horn ipsilateral to the ovary containing CL. On D25, embryonic survival was determined based on detection of embryonic vesicle with heartbeat by ultrasound scan. Values are presented as means. Pregnancy rate was affected by group (*P* = 0.06). Orthogonal contrast was used for comparisons between groups (C1: Control vs. ULF-D1; C2: ULF-D4 vs. ULF-D7; C3: Control & ULF-D1 vs. ULF-D4 & ULF-D7)

In this experiment, ULF procedure at selected time points was performed only once instead of thrice, because as verified in Exp. 1, sequential ULF resulted in no further modifications on the composition of subsequent ULFs. Furthermore, by performing a single ULF collection with low volume of DMBPS (30 mL), we expected to minimize eventual disruptions caused on uterine epithelia due the flushings. Also, ULF collection in all selected days (D1 + D4 + D7-group) had not cumulative effect on the ULF-D7.5, so this group was not included in the design. Pregnancy rate was affected by group (*P* = 0.06). Pregnancy rates were similar (*P* > 0.1) between Control (62.5%) and ULF-D1 group (60%) and between ULF-D4 (29.4%) and ULF-D7 (37.5%) groups. Pregnancy rate of Control & ULF-D1 (61.3% [19/31]) was lower (*P* = 0.03) than ULF-D4 & ULF-D7 (33.3% [11/33]), Fig. 5. Altogether, these data revealed that pregnancy rate was not affected by ULF collection when it was performed on D1, but it was impaired when ULF collection was performed on D4 or D7.

## Discussion

It is widely accepted that a biochemically well-defined uterine luminal environment (i.e., the histotroph) is required for successful pre-implantational embryo development in cattle. Moreover, developmental needs of the growing embryo/conceptus are expected to change over time. Thus, specific mechanisms must be in place to take in effect the dynamic changes needed to generate and modify the luminal molecular composition. More importantly, because incidence of embryo mortality is disproportionately elevated during this stage of gestation, it is reasonable to assume that mortality is associated with an inadequate environment. However, luminal composition that would support or suppress embryo development has not been determined. The aim of the present study was to disturb the histotroph composition through the removal of ULF to evaluate the dynamics of restoration and the effects of a disturbed uterine environment on the pregnancy outcome. We expected that uterine flushings would remove molecules that are critical for embryo development, generating an impoverished environment that would compromise development. We further anticipated that disturbances that were elicited closer in time to the moment of embryo transfer would be more detrimental, and perhaps fatal, to embryo survival. We found that uterine flushings conducted at early diestrus (D4 and D7) actually increased the total protein content in the ULF samples collected at time of ET (D7.5). Interestingly, one protein that was dramatically increased in response to ULF was albumin, the most abundant protein in serum. This supports the idea that collecting ULF caused influx of blood proteins to the uterine lumen, consequently causing a dramatic change in its composition. Alterations were most obvious in animals whose ULF were performed at 0.5 d and 3.5 d before ET, but not in animals whose ULF were performed 6.5 d before ET, indicating that it takes a relatively long time (i.e., at least 4 d) for the uterine environment to be restored after it is disturbed. Pregnancy rates to ET were consistent with the time dynamics of recovery after ULF. Indeed, ULF at time points closer to the ET (i.e. D4-ULF and D7-ULF groups) generated poorer pregnancy. Remarkably, even when ULF was conducted just 12 h prior to embryo transfer, a proportion of recipients was still able to maintain their pregnancies. This suggests that approximately one third of pregnancies will be maintained to d 25 of gestation even in a severely altered luminal environment. In contrast, it was also noteworthy that even when three embryos were transferred, approximately one third of the recipients in the control, non-manipulated group were not able to support embryo development. This indicates that there are situations in that the recipient is incompetent to maintain a gestation. Altogether, our data provide original, clear indication that recipients have varying abilities to sustain pregnancies, and embryos have varying resilience to thrive in uterine environments of very distinct quality.

Modifications on the native uterine luminal environment were evidenced by an increase on total protein quantity of ULF sampled at time of ET (D7.5) when uterine flushings were performed 0.5 to 3.5 d earlier. By conducting sequential uterine flushings we expected to deprive this specific microenvironment, decreasing the protein content according to the number of ULF collections performed. However, in fact, the abundance of proteins evaluated on the sequential ULFs sampled was unchanged. Rather than this, alterations promoted on total protein concentrations and abundance of albumin was positively influenced by samples containing blood (Fig. 2). Therefore, the effect of ULF collection on impoverishing the uterine luminal protein was probably disguised by the influx of blood proteins, such as albumin, that alone constitutes 35% to 50% of total serum protein [28]. While albumin is a normal component of uterine fluid in cattle [29], it is found in much lower concentrations in the uterine fluid (0.88 to 0.98 g/dL) than in serum samples (2.11 to 2.36 g/dL; Alavi-Shoushtari et al. [30]). Contamination by plasma and interstitial fluid is a common issue when uterine flushings are performed in vivo, transcervically in cattle [29, 31]. Even a ULF sample classified as clear by subjective evaluation contained substantial number of erythrocytes (50,400 to 4,940,000 cells/mL) as determined by hemocytometer counts [29]. Thus, the ULF collection conducted in our study clearly disrupted the native composition of the intrauterine milieu, mainly by contaminating the histotroph with plasma components.

It takes at least 4 d for total protein content and albumin concentrations in the ULF to be restored after ULF collection. To the best of our knowledge, this is the first time that dynamics of ULF re-composition after a designed disturbance was examined in cattle, in vivo. Using models that sample a given animal only in a specific, single day, others have shown the dynamic nature of histotroph changes across the estrous cycle [12–17]. The unique luminal uterine milieu seems to be maintained by dynamically active boundaries, collectively referred as blood-uterine lumen barrier [32]. Accordingly, transport and permeability properties of this barrier are influenced by factors such as steroid hormones. In rats treated with estrogen, the blood-uterine lumen barrier exhibited a selective permeability according to the biochemical nature and molecular weight of radioactive substances injected (i.e., urea, sucrose, insulin or bovine serum albumin) [33]. Furthermore, using a regimen of steroid treatment in rats, at the P4 dominance state, uterine glands showed fluid absorptive ability, while at E2 dominance state, the ability became secretory, rather than absorptive [34, 35]. Thus, during the initial diestrus, we speculate that the sex-steroid exposure may play an important role to timely regulate the restoration of the uterine luminal condition. Under the expected condition of early diestrus, i.e. progressive increase of circulating P4 concentrations, at least 4 d was necessary before the uterine luminal condition was restored.

Perturbations on the composition of ULF decreased fertility to ET. Consistent with the degree of alterations measured in the ULF at D7.5 on Exp. 1, pregnancy rates were poorer when ULF collection was conducted at time points closer to D7.5 (Fig. 5). Accordingly, ULF collection performed at D4 or D7 reduced about 50% the pregnancy maintenance to d 25, but fertility was not impacted by collection of ULF performed on D1. Interestingly, collection of ULF conducted just 12 h prior to embryo transfer, did not completely abolish pregnancy. This indicates that the native uterine milieu is permissive to deviations, although pregnancy establishment is hampered. Probably, disturbed luminal conditions exceeded the limits of tolerance of embryos in a large proportion of recipient submitted to flushing on D4 and D7, increasing the frequency of embryonic losses. Complacency of uterine luminal condition from D4-ULF and D7-ULF to embryonic survival may be also related to the similarity between the proteins found in the uterine luminal fluid and serum. In this regard, early electrophoretic evaluation, revealed that uterine fluid consisted mainly of serum proteins and a small amount of uterine-specific proteins [36, 37]. Despite of gross similarities, more recent proteomic analysis demonstrated that many plasma proteins identified in the uterine fluid had significantly different concentrations than in the plasma [38]. Thus, while suboptimal uterine condition produced by ULF-D4 and ULF-D7 is still compatible with embryonic survival, a well-defined and balanced intrauterine condition is probably required for optimal pregnancy rates. Finally, it is noteworthy, that even when three embryos were transferred, the incidence of embryonic losses in the Control group reached 37.5%, which is similar to previous reports [6–8]. This supports the concept that despite increasing the odds of embryo survival, poor uterine receptivity limited pregnancy success.

## Conclusions

This study showed that subtle perturbations of the uterine environment, such as those caused by a single, low-volume collection of ULF, profoundly disturbs intrauterine composition and pregnancy success. However, after at least 4 d of the insult, the uterus has the capacity to recover its composition and the functional capacity to carry post-implantation gestation.

## Abbreviations

ANOVA: Analysis of variance
CL: Corpus luteum
CV: Coefficient of variation
DMPBS: Dulbecco’s modified phosphate buffered saline
E2: Estradiol
ET: Embryo transfer
GnRH: Gonadotrophin releasing hormone
INFT: Interferon-tau
LSMEANS: Least Square Means
P4: Progesterone
PGF_2_α: Prostaglandin F_2_α
RIA: Radioimmunoassay
SDS-PAGE: Sodium dodecyl sulfate polyacrylamide gel electrophoresis
SEM: Standard error of the mean
ULF: Uterine luminal flushings
